# Genetic and Chemical Activation of TFEB Mediates Clearance of Aggregated α-Synuclein

**DOI:** 10.1371/journal.pone.0120819

**Published:** 2015-03-19

**Authors:** Kiri Kilpatrick, Yimeng Zeng, Tommy Hancock, Laura Segatori

**Affiliations:** 1 Department of Chemical and Biomolecular Engineering, Rice University, Houston, Texas, United States of America; 2 Department of Bioengineering, Rice University, Houston, Texas, United States of America; 3 Department of Biochemistry and Cell Biology. Rice University, Houston, Texas, United States of America; The Tokyo Metropolitan Institute Medical Science, JAPAN

## Abstract

Aggregation of α-synuclein (α-syn) is associated with the development of a number of neurodegenerative diseases, including Parkinson’s disease (PD). The formation of α-syn aggregates results from aberrant accumulation of misfolded α-syn and insufficient or impaired activity of the two main intracellular protein degradation systems, namely the ubiquitin-proteasome system and the autophagy-lysosomal pathway. In this study, we investigated the role of transcription factor EB (TFEB), a master regulator of the autophagy-lysosomal pathway, in preventing the accumulation of α-syn aggregates in human neuroglioma cells. We found that TFEB overexpression reduces the accumulation of aggregated α-syn by inducing autophagic clearance of α-syn. Furthermore, we showed that pharmacological activation of TFEB using 2-hydroxypropyl-β-cyclodextrin promotes autophagic clearance of aggregated α-syn. In summary, our findings demonstrate that TFEB modulates autophagic clearance of α-syn and suggest that pharmacological activation of TFEB is a promising strategy to enhance the degradation of α-syn aggregates.

## Introduction

Parkinson’s disease (PD) is the most prevalent neurodegenerative movement disorder. It is characterized by the accumulation of proteinaceous cytoplasmic inclusions (Lewy bodies) in dopaminergic neurons [1]. The major component of Lewy bodies is α-synuclein (α-syn) [2], a natively unfolded 140 amino-acid protein with high propensity to misfold and aggregate [3]. The role of α-syn in the development of PD has been extensively investigated and evidence points to a correlation between α-syn misfolding and aggregation and the progression of PD pathogenesis [4].

The ubiquitin-proteasome system (UPS) provides the primary route for degradation of misfolded α-syn [5]. A reduction in proteasome activity appears to be linked to the accumulation of misfolded and aggregated α-syn [6] and genetic mutations in UPS components have been associated with neurodegeneration in familial forms of PD [7]. Primarily responsible for mediating the degradation of long-lived proteins by the lysosome [8], autophagy also plays a key role in promoting clearance of misfolded and aggregated α-syn [9,10]. The autophagy pathway and the UPS mediate coordinated and complementary roles, which become particularly critical under conditions of proteotoxic stress [11]. Not surprisingly, recent evidence suggests that adaptive or pharmacologically induced activation of autophagy is likely to play a key role in maintaining protein homeostasis when the UPS capacity is insufficient or compromised [12–14].

Macroautophagy mediates clearance of protein aggregates. It involves cargo sequestration into autophagosomes, fusion of autophagosomes with lysosomes leading to formation of autophagolysosomes, and cargo degradation by lysosomal hydrolases [15]. In addition to macroautophagy (hereafter referred to as autophagy), cytoplasmic material can be delivered to the lysosome for degradation through chaperone-mediated autophagy (CMA), which involves selective translocation of soluble cytoplasmic proteins into the lysosome [16], or through microautophagy, which involves non-selective engulfment of cytoplasmic cargo into the lysosome [17]. Impairment of autophagy is often linked to accumulation of proteinaceous aggregates and neurodegeneration [18].

Impairment of autophagy has been observed in association with development of PD. Autophagic activity generally declines with age and autophagic markers are found to be decreased in *post mortem* brain tissues from PD patients [19,20], suggesting a link between autophagic clearance and accumulation of aggregated α-syn. In addition, α-syn transgenic mice are characterized by lowered autophagic activity and progressive neurodegeneration [20]. These phenotypes can be rescued by upregulating essential components of the autophagy system, such as Beclin-1, Atg7, and Rab1a [20–23]. Pathogenic variants of α-syn may also block protein translocation into the lysosome and reduce α-syn degradation by CMA [10]. Interestingly, evidence suggests an increased susceptibility to α-syn aggregation in diseases characterized by lysosomal dysfunction, such as Gaucher’s and Niemann-Pick diseases, underscoring the role of the lysosomes in mediating autophagic clearance of α-syn [24,25]. Taken together, these studies point to the important role of autophagy in mediating clearance of α-syn and suggest that enhancement of autophagic clearance could ameliorate the phenotypes associated with accumulation of α-syn aggregates, thereby providing a therapeutic strategy for the treatment of PD [26].

Novel insights into the mechanisms of autophagy regulation have emerged with the recent discovery that the transcription factor EB (TFEB) controls the coordinated activation of the CLEAR (Coordinated Lysosomal Expression and Regulation) network [27,28]. TFEB regulates lysosome biogenesis [28,29] as well as autophagosome formation and autophagosome-lysosome fusion, thereby promoting cellular clearance [27]. Overexpression of TFEB was found to decrease the accumulation of polyglutamine-containing huntingtin aggregates in a rat striatal cell model of Huntington’s disease [27] and reduce huntingtin aggregate formation in Neuro2a cells subjected to oxidative stress [30]. Overexpression of TFEB was also shown to reduce neurodegeneration in *in vitro* and *in vivo* models of PD by restoring lysosome levels and increasing autophagic clearance [31,32]. The reduction in oligomeric α-syn species observed in transgenic rats in which TFEB is overexpressed also suggests that TFEB plays a key role in reducing the accumulation of aggregated α-syn. However, the molecular mechanisms underlying TFEB-mediated clearance of aggregated α-syn remain uncharacterized.

Based on evidence that α-syn misfolding and aggregation is often linked to inefficient function of quality control mechanisms that regulate degradation of aberrant proteins and that TFEB is a master regulator of lysosomal biogenesis and autophagy, we hypothesized that TFEB activation could prevent accumulation of α-syn aggregates by enhancing autophagic clearance. We tested this hypothesis by using a human neuroglioma stable cell line that accumulates aggregated α-syn [33] and demonstrated that overexpression of TFEB reduces the accumulation of aggregated α-syn. Specifically, we provide evidence that the reduced accumulation of α-syn aggregates correlates with TFEB activation and with upregulation of the CLEAR network and the autophagy system. We also show that chemical activation of TFEB using 2-hydroxypropyl-β-cyclodextrin (HPβCD) mediates autophagic clearance of aggregated α-syn. These results support the role of TFEB as a therapeutic target for the treatment of PD and potentially other neurodegenerative diseases characterized by protein aggregation.

## Results and Discussion

### TFEB promotes clearance of α-syn aggregates

**Fig 1:**
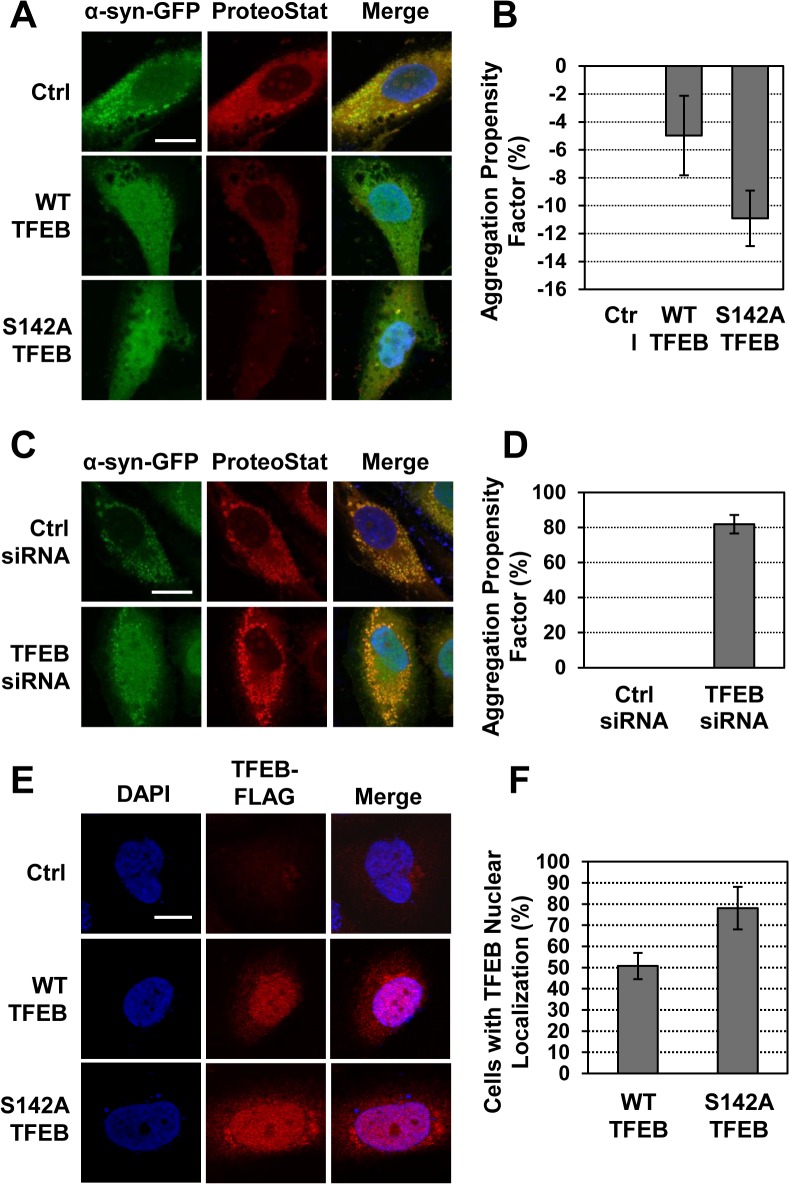
TFEB overexpression results in reduced accumulation of α-syn aggregates. **a)** Fluorescence microscopy analyses of H4/α-syn-GFP cells transduced to express TFEB-3xFLAG or S142A TFEB-3xFLAG. Images of α-syn-GFP fluorescence (green, column 1) and aggregates, detected using the ProteoStat dye (red, column 2), were merged (column 3) and analyzed using NIH ImageJ software. Representative images are reported. Scale bar represents 20 μm. **b)** Total protein aggregation in H4/α-syn-GFP cells transduced to express TFEB-3xFLAG or S142A TFEB-3xFLAG. Total protein aggregation was quantified by measuring binding of the ProteoStat dye by flow cytometry. The aggregation propensity factor (APF) was calculated as described in Methods and normalized to TFEB mRNA expression. Data are reported as mean ± SD (n ≥ 3; p < 0.01). **c)** Fluorescence microscopy analyses of H4/α-syn-GFP cells treated with control siRNA or *TFEB* siRNA. Images were analyzed as described in (a). Representative images are reported. Scale bar represents 20 μm. **d)** Total protein aggregation in H4/α-syn-GFP treated with control siRNA or *TFEB* siRNA. Total protein aggregation was quantified as described in (b). Data are reported as mean ± SD (n ≥ 3; p < 0.01). e) Immunofluorescence microscopy analyses of TFEB subcellular localization in H4/α-syn-GFP cells transduced to express TFEB-3xFLAG or S142A TFEB-3xFLAG. TFEB nuclear localization was monitored using a FLAG-specific antibody and DAPI nuclear stain. Colocalization of DAPI (blue, column 1) and TFEB-3xFLAG (red, column 2) is shown in purple (column 3). Representative images are reported. Scale bar represents 10 μm. **f)** Percentage of cells transduced as described in (e) presenting TFEB nuclear localization. Representative fields containing 50–100 cells were analyzed (p < 0.05).

To investigate the role of TFEB in regulating the accumulation of α-syn aggregates, we used neuroglioma cells stably transfected for the expression of α-syn fused to GFP (H4/α-syn-GFP) [34]. The use of α-syn-GFP as a valid reporter for disease-associated phenotypes has been previously established [35–37]. We first overexpressed TFEB fused to a FLAG tag (TFEB-3xFLAG) in H4/α-syn-GFP cells by retroviral transduction and evaluated the presence of α-syn-GFP aggregates. α-syn aggregates were evaluated as previously demonstrated [34,35] by monitoring GFP fluorescence and binding of the ProteoStat dye, a 488-nm excitable red fluorescent molecule that specifically interacts with denatured proteins within protein aggregates [38]. Fluorescence microscopy images of α-syn aggregation in control (non-transduced) H4/α-syn-GFP cells show punctate GFP fluorescence (Fig. 1A, column 1, green) that colocalizes with the ProteoStat dye signal (column 2, red), as shown in merged images (column 3, yellow). In H4/α-syn-GFP cells that were transduced to induce TFEB overexpression, we observed diffuse GFP fluorescence that does not colocalize with the ProteoStat dye signal, suggesting that increasing TFEB expression reduces the accumulation of α-syn aggregates.

TFEB localizes predominantly in the cytoplasm of resting cells and translocates into the nucleus upon activation [28]. To investigate whether the reduction of α-syn aggregates depends on activation of TFEB, we evaluated α-syn aggregation in cells expressing a TFEB variant (TFEB-S142A) that localizes preferentially in the nucleus [27]. Overexpression of S142A TFEB via retroviral transduction of H4/α-syn-GFP cells with TFEB-S142A-3xFLAG was found to result in diffuse GFP signal and, importantly, to reduce ProteoStat dye binding to a larger extent than overexpression of wild type TFEB (WT TFEB). We also observed complete lack of co-localization of GFP and ProteoStat signal, suggesting that activation of TFEB prevents accumulation of aggregated α-syn (Fig. 1A, row 3). The decrease in protein aggregation observed upon overexpression of WT TFEB is likely due to activation of TFEB induced by accumulation of α-syn aggregates, similar to what was previously reported in disease cells presenting deposition of storage material [28].

To confirm that TFEB expression and activation levels mediate reduction in protein aggregation, we quantified the extent of total protein aggregation by evaluating the aggregation propensity factor (APF; see Methods) of H4/α-syn-GFP cells overexpressing WT TFEB or S142A TFEB relative to non-transduced control cells (Fig. 1B). APF values were corrected by *TFEB* mRNA expression levels (evaluated by quantitative RT-PCR (qRT-PCR)) to account for differences in transduction efficiencies. We observed a decrease in total protein aggregation in H4/α-syn-GFP cells expressing WT TFEB compared to control cells (APF = −5.0%), which further decreased upon overexpression of S142A TFEB (APF = −10.9%), suggesting that TFEB prevents accumulation of aggregated proteins. Importantly, the changes in total protein aggregation observed upon overexpression of WT and S142A TFEB recapitulate the decrease in α-syn aggregates observed by confocal microscopy.

To directly evaluate the role of TFEB in regulating the accumulation of α-syn aggregates, we monitored the extent of α-syn aggregation upon silencing *TFEB* expression. Small interfering RNA (siRNA) was used to silence *TFEB* expression in H4/α-syn-GFP cells and the accumulation of α-syn-GFP aggregates was analyzed by confocal microscopy. Treatment with *TFEB* siRNA resulted in 60% reduction in *TFEB* expression compared to cells transfected with control siRNA, as determined by qRT-PCR. Microscopy images of H4/α-syn-GFP cells treated with *TFEB* siRNA presented diffuse GFP fluorescence and strong colocalization of GFP and ProteoStat dye signals (Fig. 1C). Interestingly, the APF of cells treated with *TFEB* siRNA was found to increase to 81.9% compared to cells treated with control siRNA confirming that TFEB plays a key role in regulating the accumulation of α-syn aggregates (Fig. 1D).

To confirm the correlation between TFEB activation and accumulation of aggregated α-syn, we evaluated TFEB nuclear localization in H4/α-syn-GFP cells under conditions observed to induce reduction in α-syn aggregation. H4/α-syn-GFP cells transduced to overexpress WT TFEB-3xFLAG or TFEB-S142A-3xFLAG were analyzed using a FLAG-specific antibody and DAPI nuclear stain (Fig. 1E). Immunofluorescence analyses revealed complete lack of TFEB signal in control cells lacking transgene expression, as expected, and nuclear localization of TFEB in cells overexpressing WT TFEB or S142A TFEB, as indicated by colocalization of anti-FLAG and DAPI signals. TFEB nuclear localization was quantified by calculating the fraction of transduced cells (FLAG-positive) that present nuclear localization of TFEB (Fig. 1F). TFEB was found to localize in the nucleus of 50.7% of H4/α-syn-GFP cells expressing WT TFEB and in 78.1% of H4/α-syn-GFP cells expressing S142A TFEB suggesting a correlation between TFEB activation and accumulation of aggregated α-syn.

TFEB activation was also confirmed by testing the expression of genes that are known targets of TFEB and are upregulated upon TFEB activation. The mRNA levels of representative genes, namely *GBA* (Glucocerebrosidase), *HEXA* (Hexosaminidase A), and *LAMP1* (Lysosome-associated membrane glycoprotein 1), were monitored by qRT-PCR and were found to be significantly upregulated upon overexpression of WT and S142A TFEB (S1 Fig.).

These results, taken together, indicate that the reduction in α-syn aggregation observed upon overexpression of TFEB in H4/α-syn-GFP cells correlates with the extent of TFEB expression and activation.

### TFEB-mediated clearance of α-syn involves activation of autophagy

**Fig 2:**
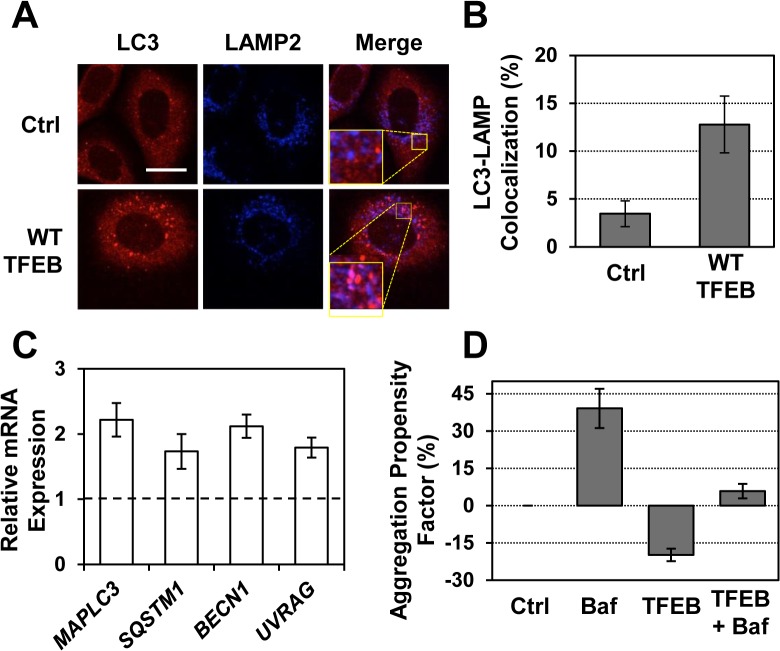
TFEB mediates reduction of α-syn aggregates by inducing autophagic clearance. a) Immunofluorescence microscopy analyses of LC3 and LAMP2 in H4/α-syn-GFP cells transduced to express TFEB-3xFLAG. Colocalization of LC3 (red, column 1) and LAMP2 (blue, column 2) is shown in purple (column 3). Representative images are reported. Scale bar represents 20 μm. **b)** Quantification of LC3- LAMP2 colocalization was calculated using randomly selected images containing 30–50 cells obtained from three independent experiments (p < 0.001). **c)** Relative mRNA expression levels of representative genes involved in the autophagy pathway in H4/α-syn-GFP cells transduced to overexpress TFEB. *MAPLC3*, *SQSTM1*, *BECN1*, and *UVRAG* mRNA expression levels were obtained by qRT-PCR, corrected for the expression of the housekeeping genes *GAPDH* and *ACTB*, and normalized to those of untreated cells (dashed line). Data are reported as mean ± SD (n ≥ 3; p < 0.05, *p < 0.01). **d)** Total protein aggregation in H4/α-syn-GFP cells transduced to express TFEB-3xFLAG and treated with bafilomycin (100 nM). Total protein aggregation was quantified by measuring binding of the ProteoStat dye by flow cytometry. The APF was calculated as described in the Methods. Data are reported as mean ± SD (n ≥ 3; p < 0.05).

To determine whether the reduced accumulation of α-syn aggregates observed upon TFEB overexpression in H4/α-syn-GFP cells results from TFEB-mediated activation of autophagy and autophagic clearance of α-syn aggregates, we tested a series of autophagic markers. We first monitored LC3 (microtubule-associated light chain protein 3) in H4/α-syn-GFP cells overexpressing TFEB. LC3 is recruited to autophagosomal membranes [39] and is widely used as a marker of autophagy induction [40]. H4/α-syn-GFP cells overexpressing TFEB were first analyzed by confocal microscopy using an LC3-specific antibody to visualize LC3 structures (Fig. 2A, column 1). As expected, we observed a diffuse LC3 signal in control cells, indicating basal autophagic activity. The formation of punctate LC3 structures in cells overexpressing TFEB indicates the accumulation of autophagosomes. These results are consistent with previous reports demonstrating that TFEB activation results in enhanced formation of autophagosomes [27].

To investigate the correlation between TFEB activation and autophagic clearance under conditions that result in reduced accumulation of aggregated α-syn, we evaluated the formation of autophagolysosomes [15]. Autophagolysosomes were detected by evaluating the extent of colocalization between LC3, an autophagosomal membrane protein, and LAMP2, a lysosomal membrane protein [40]. H4/α-syn-GFP cells overexpressing TFEB were incubated with antibodies specific for LC3 and LAMP2 and analyzed by confocal microscopy (Fig. 2A). Overexpression of TFEB resulted in enhanced colocalization of LC3 (column 1, red) and LAMP2 (column 2, blue), as shown in the merged images (column 3, purple), compared to control cells. The extent of colocalization was quantified by calculating the percentage of pixels exhibiting positive correlation between LC3 and LAMP2 (Fig. 2B; see Methods). LC3-LAMP2 colocalization was found to increase from 3.5% in control cells to 12.8% in cells overexpressing TFEB.

To confirm that TFEB-mediated reduction in α-syn aggregates parallels activation of autophagy, we tested the expression of genes involved in different steps of the autophagy pathway. H4/α-syn-GFP cells were transduced to express TFEB and the mRNA levels were measured by qRT-PCR (Fig. 2C). TFEB overexpression resulted in significant upregulation of *MAPLC3* (LC3; 2.2-fold), which is involved in the formation of autophagic vesicles as described above, *SQSTM1* (p62; 1.7-fold), which is involved in cargo recognition, and *BECN1* (Beclin-1; 2.1-fold) and *UVRAG* (UV Radiation Resistance-Associated Gene; 1.8-fold), which are required for the formation of autophagosomes. Interestingly, *MAPLC3*, *SQSTM1*, and *UVRAG* are known to be direct targets of TFEB [27].

To directly assess whether the decrease protein aggregates in H4/α-syn-GFP cells depends on TFEB mediated activation of autophagy, we evaluated the extent of protein aggregation in cells overexpressing TFEB and treated to block the autophagic flux. Specifically, H4/α-syn-GFP cells transduced with TFEB-3xFLAG were treated with bafilomycin, an inhibitor of vacuolar H^+^ ATPase (V-ATPase) activity that prevents fusion of autophagosomes with lysosomes [41]. Preliminary studies were conducted to evaluate the optimal bafilomycin treatment conditions to achieve inhibition of autophagy without causing cell death, which would preclude accurate investigation of protein aggregation. As expected, bafilomycin treatment (100 nM) increased ProteoStat dye binding (APF = 39.1%) compared to control cells (Fig. 2D). We also observed an increase in ProteoStat dye binding upon addition of bafilomycin to cells overexpressing TFEB (APF = 5.8%) compared to cells overexpressing TFEB and not treated with bafilomycin (APF = −20.7%). These results indicate that inhibition of downstream steps of the autophagy pathway (i.e., autophagolysosome formation) prevents TFEB-mediated reduction in protein aggregation.

In summary, these results demonstrate that TFEB activation reduces accumulation of α-syn aggregates in neuroglioma cells overexpressing α-syn-GFP and that autophagy plays a key role in TFEB-mediated clearance of aggregated α-syn.

### Chemical activation of TFEB results in clearance of aggregated α-syn

To further verify that TFEB mediates clearance of α-syn aggregates we tested chemical activation of TFEB in H4/α-syn-GFP cells using 2-hydroxypropyl-β-cyclodextrin (HPβCD) [42], which was recently reported to function as a chemical activator of TFEB. Particularly, HPβCD treatment was shown to induce nuclear translocation of TFEB, upregulation of the CLEAR network, and increase in autophagic clearance [43]. Previous studies showed that methyl-β-cyclodextrin (MβCD) reduces α-syn aggregation in rat neuroblastoma cells and in transgenic mice overexpressing α-syn [44]; however, the molecular mechanisms underlying MβCD-induced reduction in α-syn aggregation are unclear.

**Fig 3:**
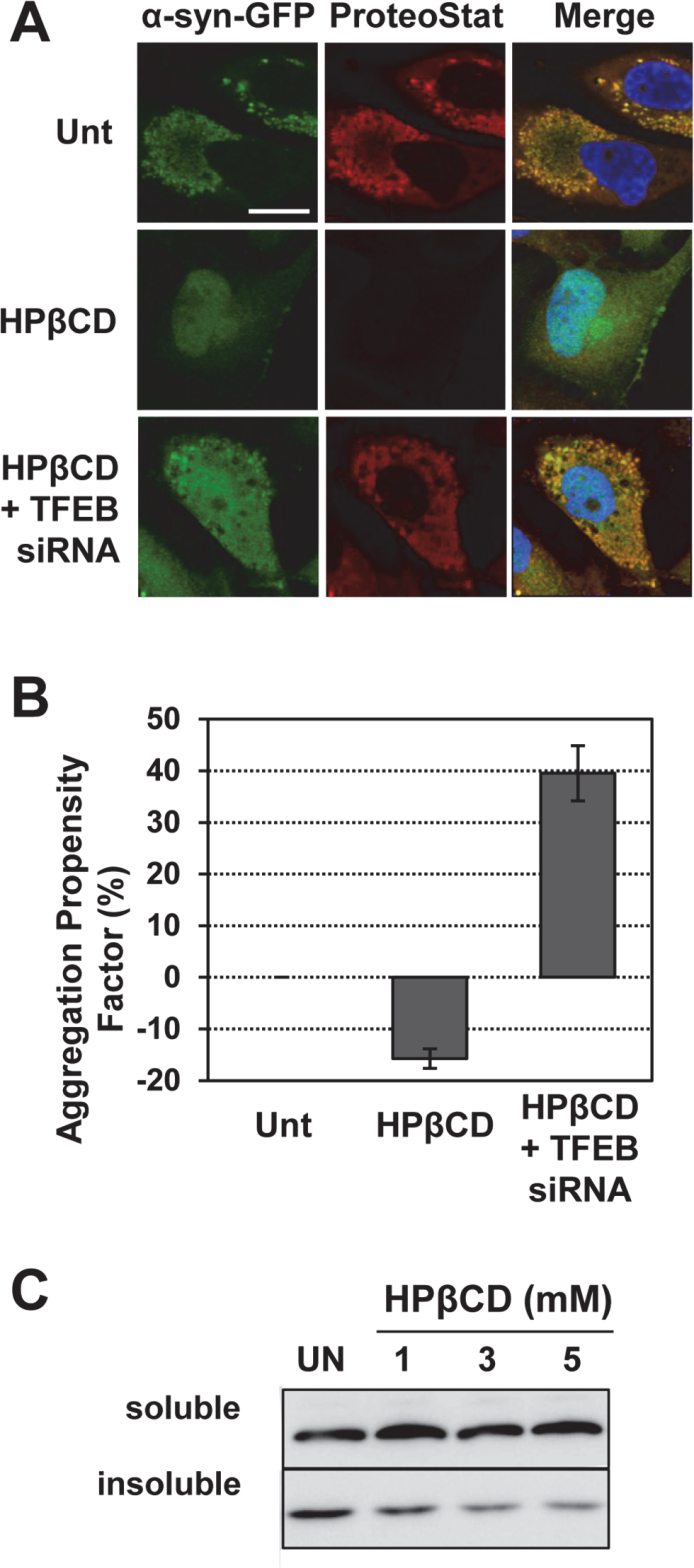
HPβCD treatment results in clearance of α-syn aggregates. **a)** Fluorescence microscopy analyses of H4/α-syn-GFP cells treated with HPβCD (1 mM) for 24 h with or without *TFEB* siRNA. Images of α-syn-GFP fluorescence (green, column 1) and aggregates, detected using the ProteoStat dye (red, column 2), were merged (column 3) and analyzed using NIH ImageJ software. Representative images are reported. Scale bar represents 20 μm. **b)** Total protein aggregation in H4/α-syn-GFP cells treated with control siRNA or *TFEB* siRNA for 48 h and with or without HPβCD (1 mM) for 24 h. Total protein aggregation was quantified by measuring binding of the ProteoStat dye by flow cytometry. The aggregation propensity factor (APF) was calculated as described in the Methods. Data are reported as mean ± SD (n ≥ 3; p<0.05). **c)** Western blot analyses of α-syn in Triton X-100 soluble and insoluble fraction of H4/α-syn-GFP cells treated with HPβCD (1mM, 3mM and 5mM).

We first evaluated the formation of α-syn aggregates in H4/α-syn-GFP cells treated with HPβCD by monitoring GFP and ProteoStat dye fluorescence. Preliminary studies were conducted using a range of HPβCD concentrations to determine the optimal HPβCD dosage that reduces α-syn aggregates without altering cell viability (not shown). Cell treatment with HPβCD (1 mM) resulted in the appearance of diffuse GFP fluorescence, reduction in ProteoStat dye binding, and lack of colocalization between GFP and ProteoStat dye signals (Fig. 3A, row 2), suggesting that HPβCD treatment prevents accumulation of α-syn aggregates and recapitulating the results observed upon genetic activation of TFEB (Fig. 1). HPβCD treatment under these conditions was confirmed not to induce activation of early or late apoptosis, as evaluated by monitoring Annexin V and PI binding (S2 Fig.).

To directly assess whether HPβCD-induced reduction in α-syn aggregates is mediated by TFEB, we evaluated the effect of HPβCD on the accumulation of α-syn aggregates upon silencing of *TFEB* expression (Fig. 3A, row 3). We observed reappearance of punctate GFP signal, binding of ProteoStat dye, and colocalization of ProteoStat and GFP signals, indicating that TFEB mediates HPβCD-induced reduction of α-syn aggregates. Treatment with control siRNA did not alter HPβCD-induced reduction of α-syn aggregates (S3 Fig.). Flow cytometry analyses confirmed that HPβCD treatment lowers the extent of total protein aggregation (APF = −15.7%) (Fig. 3B). Silencing *TFEB* in cells treated with HPβCD, on the other hand, resulted in a dramatic increase in ProteoStat dye binding (APF = 39.6%). These results, taken together, suggest that HPβCD treatment reduces the accumulation of α-syn aggregates and that this effect is mediated by TFEB. α-syn-GFP aggregation was also tested by evaluating the relative accumulation of α-syn in Triton X-100 soluble and insoluble protein fractions of H4/α-syn-GFP cells treated with HPβCD (1mM, 3mM and 5mM) by Western blot. HPβCD treatment resulted in decrease in insoluble α-syn compared to the untreated control (Fig. 3C) in a HPβCD concentration dependent fashion (S4 Fig.), but did not affect the pool of soluble α-syn. These results are in agreement with what was observed from the microscopy and flow cytometry studies and confirm that HPβCD treatment reduces the accumulation of α-syn aggregates.

**Fig 4:**
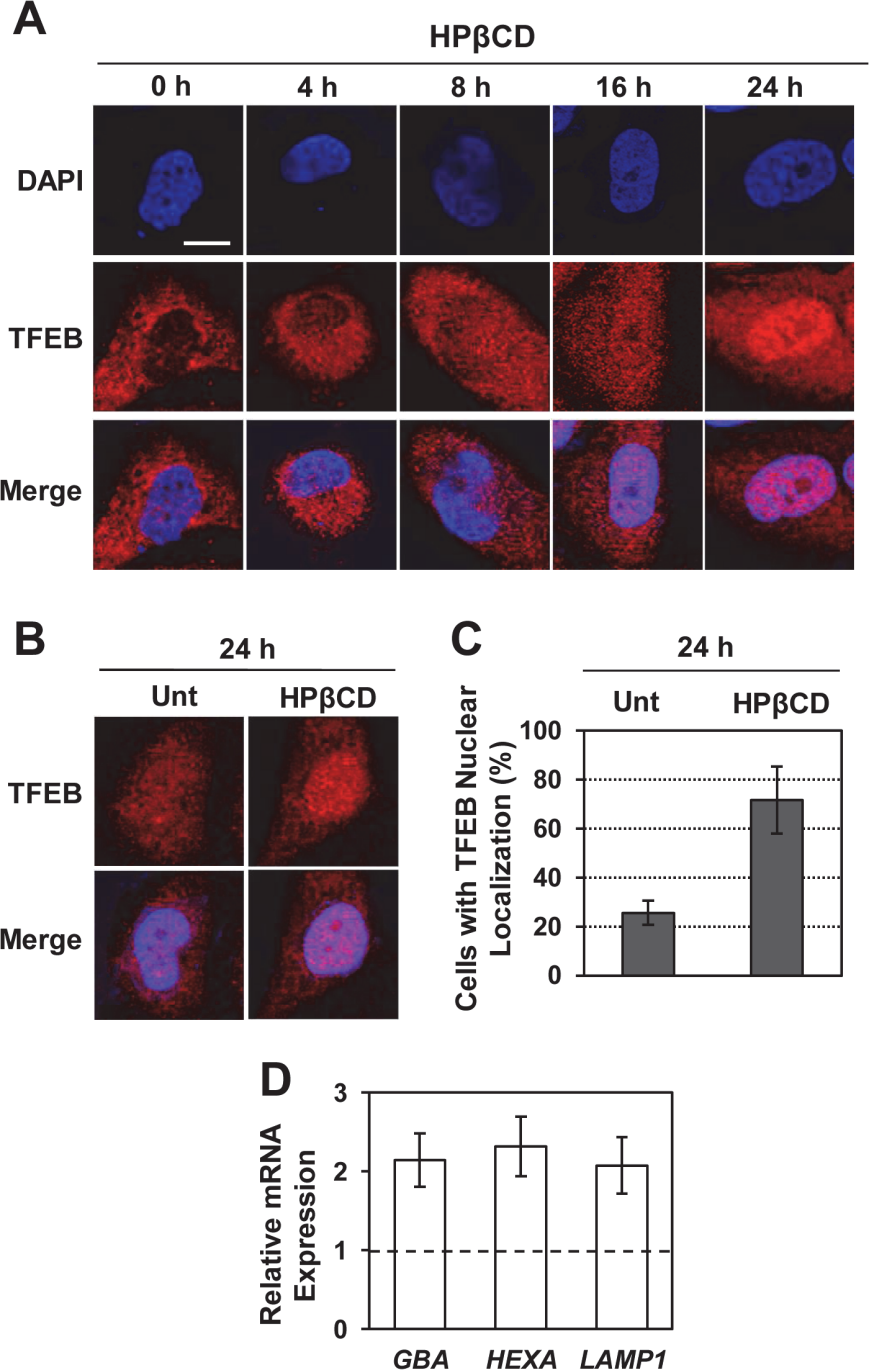
HPβCD treatment induces activation of TFEB in H4/α-syn-GFP cells. **a-b)** Immunofluorescence microscopy analysis of TFEB subcellular localization in H4/α-syn-GFP cells treated with HPβCD (1 mM). TFEB nuclear localization was monitored using a TFEB-specific antibody and DAPI nuclear stain. Colocalization of DAPI (blue, row 1) and TFEB (red, row 2) is shown in purple (row 3). Scale bar represents 10 μm. **c)** Percentage of HPβCD-treated cells presenting TFEB nuclear localization. Representative fields containing 50–100 cells were analyzed (p < 0.05). **d)** Relative mRNA expression levels of representative CLEAR network genes in H4/α-syn-GFP cells treated with HPβCD (1 mM) for 24 h. *GBA*, *HEXA*, and *LAMP1* mRNA expression levels were obtained by qRT-PCR and calculated as described in Fig. 2C. Data are reported as mean ± SD (n ≥ 3; p < 0.01).

To verify that HPβCD treatment under conditions that result in reduction of α-syn aggregates in H4/α-syn-GFP cells causes TFEB activation, we evaluated TFEB nuclear localization and expression of representative genes that are known targets of TFEB. Intracellular localization of TFEB was monitored by confocal microscopy using a TFEB-specific antibody. Microscopy images were taken at various time points after the addition of HPβCD in the culturing medium (Fig. 4A) and TFEB was found to progressively translocate into the nucleus of HPβCD-treated H4/α-syn-GFP cells. TFEB nuclear translocation, quantified by calculating the fraction of cells that present nuclear localization of TFEB, was found to increase from 25.5% to 71.6% after 24hr of HPβCD treatment (Fig. 4B and C). We also detected significant upregulation of the TFEB target genes tested (Fig. 4D), namely *GBA* (2.1-fold), *HEXA* (2.3-fold), and *LAMP1* (2.1-fold), confirming that TFEB nuclear translocation induced by HPβCD results in activation of the CLEAR network, as previously observed [42].

### Chemical activation of TFEB enhances autophagic clearance

**Fig 5:**
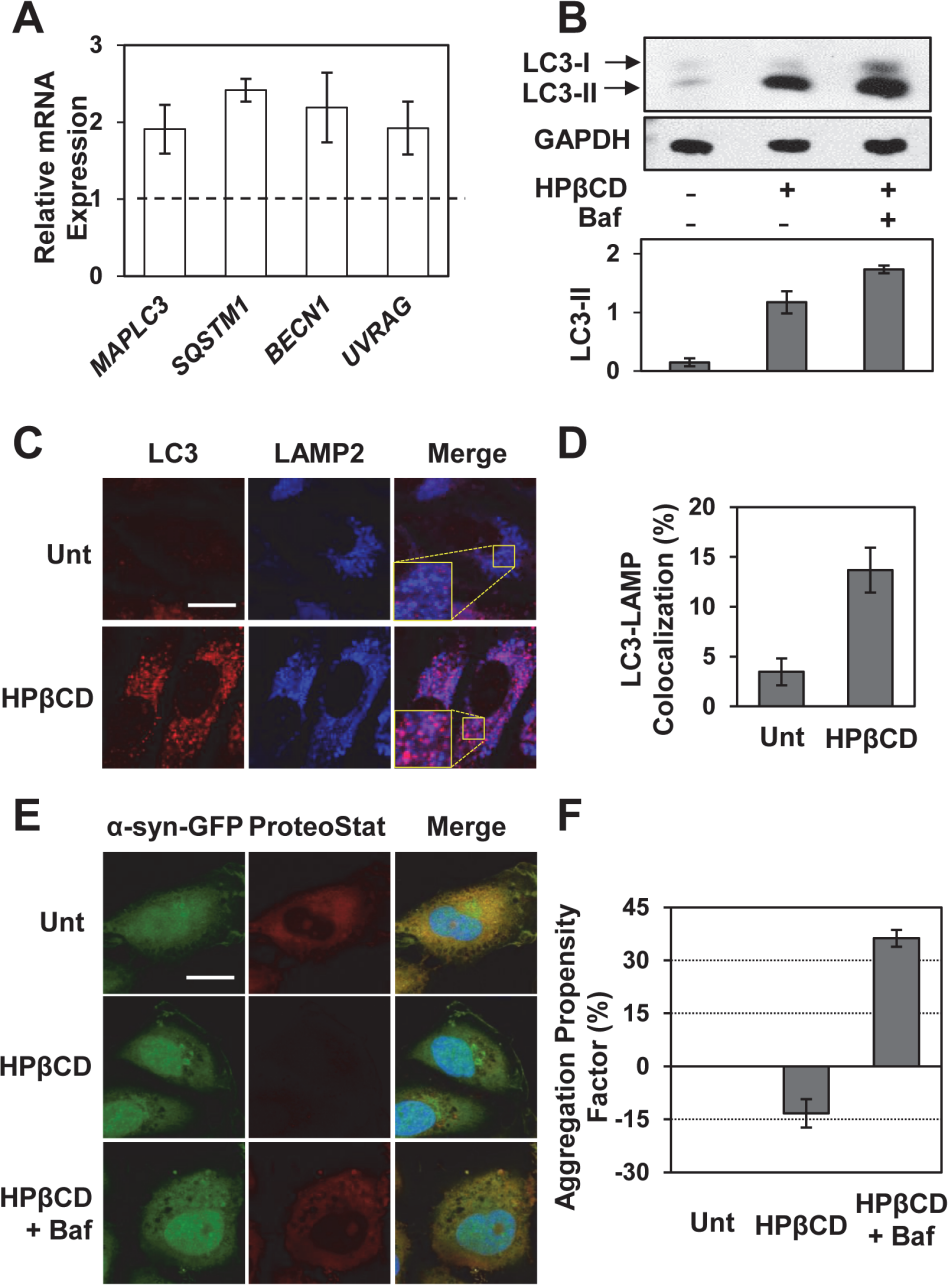
HPβCD treatment enhances autophagic clearance of α-syn aggregates in H4/α-syn-GFP cells. **a)** Relative mRNA expression levels of representative genes of the autophagy pathway in H4/α-syn-GFP cells treated with HPβCD (1 mM) for 24 h. *MAPLC3*, *SQSTM1*, *BECN1*, and *UVRAG* mRNA expression levels were obtained by qRT-PCR and calculated as described in Fig. 2C (p < 0.05). **b)** Western blot analyses of LC3 isoforms and GAPDH (used as loading control) in H4/α-syn-GFP cells treated with HPβCD (1 mM) for 24 h and quantification of LC3-II bands. Band intensities were quantified with NIH ImageJ software and corrected by GAPDH band intensities (p < 0.05) **c)** Immunofluorescence microscopy analysis of LC3 and LAMP2 in H4/α-syn-GFP cells treated with HPβCD (1 mM) for 24 h. Colocalization of LC3 (red, column 1) and LAMP2 (blue, column 2) is shown in purple (column 3). Representative images are reported. Scale bars represent 20 μm. **d)** Quantification of LC3-LAMP2 colocalization was calculated using randomly selected images containing 30–50 cells obtained from three independent experiments (p < 0.001). **e)** Fluorescence microscopy analyses of H4/α-syn-GFP cells untreated or treated with HPβCD (1 mM) and/or bafilomycin (100 nM) for 24 h. Images of α-syn-GFP fluorescence (green, column 1) and aggregates, detected using the ProteoStat dye (red, column 2), were merged (column 3) and analyzed using NIH ImageJ software. Representative images are reported. Scale bar represents 20 μm. f) Total protein aggregation in H4/α-syn-GFP cells untreated or treated with HPβCD (1 mM) and/or bafilomycin (100 nM) for 24 h. Total protein aggregation was quantified by measuring binding of the ProteoStat aggregation dye by flow cytometry. The APF was calculated as described in the Methods. Data are reported as mean ± SD (n ≥ 3; p < 0.05).

To investigate whether autophagy is involved in HPβCD-mediated reduction of α-syn aggregates in H4/α-syn-GFP cells we monitored a series of autophagic markers upon treatment with HPβCD. To confirm upregulation of the autophagy system, we first verified upregulation of representative genes involved in the autophagy pathway (Fig. 5A), namely *MAPLC3* (1.9-fold), *SQSTM1* (2.2-fold), *BECN1* (2.2-fold), and *UVRAG* (1.9-fold).

Activation of autophagy in H4/α-syn-GFP cells treated with HPβCD was also confirmed by immunoblotting of LC3 isoforms (Fig. 5B). HPβCD treatment resulted in increase in LC3-II, suggesting enhanced formation of autophagic vesicles [40]. The further increase in LC3-II levels observed in cells treated with HPβCD in the presence of the autophagy inhibitor bafilomycin, compared to cells treated only with HPβCD, indicates an increase in autophagic flux. These results suggest that HPβCD treatment induces activation of autophagy in H4/α-syn-GFP cells.

To monitor the formation of autophagosomes and autophagolysosomes, we evaluated the formation of LC3 puncta and colocalization of LC3 and LAMP2, respectively (Fig. 5C). We observed punctate LC3 structures in cells treated with HPβCD (column 1, red), indicating enhanced autophagosome formation, as well as increase in colocalization of LC3 and LAMP2 (column 2, blue) as shown in merged images (column 3, purple), indicating enhanced autophagolysosome formation. Specifically, HPβCD treatment of H4/α-syn-GFP cells resulted in a 4-fold increase in autophagolysosome formation (Fig. 5D).

These results, taken together, demonstrate that treatment of H4/α-syn-GFP cells with HPβCD, under conditions that result in TFEB activation and reduced accumulation of α-syn aggregates, activates autophagy.

To directly assess whether the decrease in α-syn aggregates observed in H4/α-syn-GFP cells treated with HPβCD depends on autophagic activity, we monitored α-syn aggregation upon inhibition of autophagy using bafilomycin. The accumulation of α-syn-GFP aggregates was examined by evaluating GFP and ProteoStat dye fluorescence in H4/α-syn-GFP cells treated with HPβCD and bafilomycin (100 nM). We found that bafilomycin prevents HPβCD-mediated reduction in accumulation of α-syn-GFP aggregates (Fig. 5D, compare HPβCD to HPβCD+bafilomycin images). Flow cytometry analyses conducted to quantify ProteoStat dye binding confirmed that bafilomycin treatment also results in an increase in total protein aggregation in HPβCD-treated H4/α-syn-GFP cells (Fig. 5F; APF = 36.3%). These results indicate that HPβCD-mediated reduction in α-syn aggregates in H4/α-syn-GFP cells depends on autophagic clearance.

**Fig 6:**
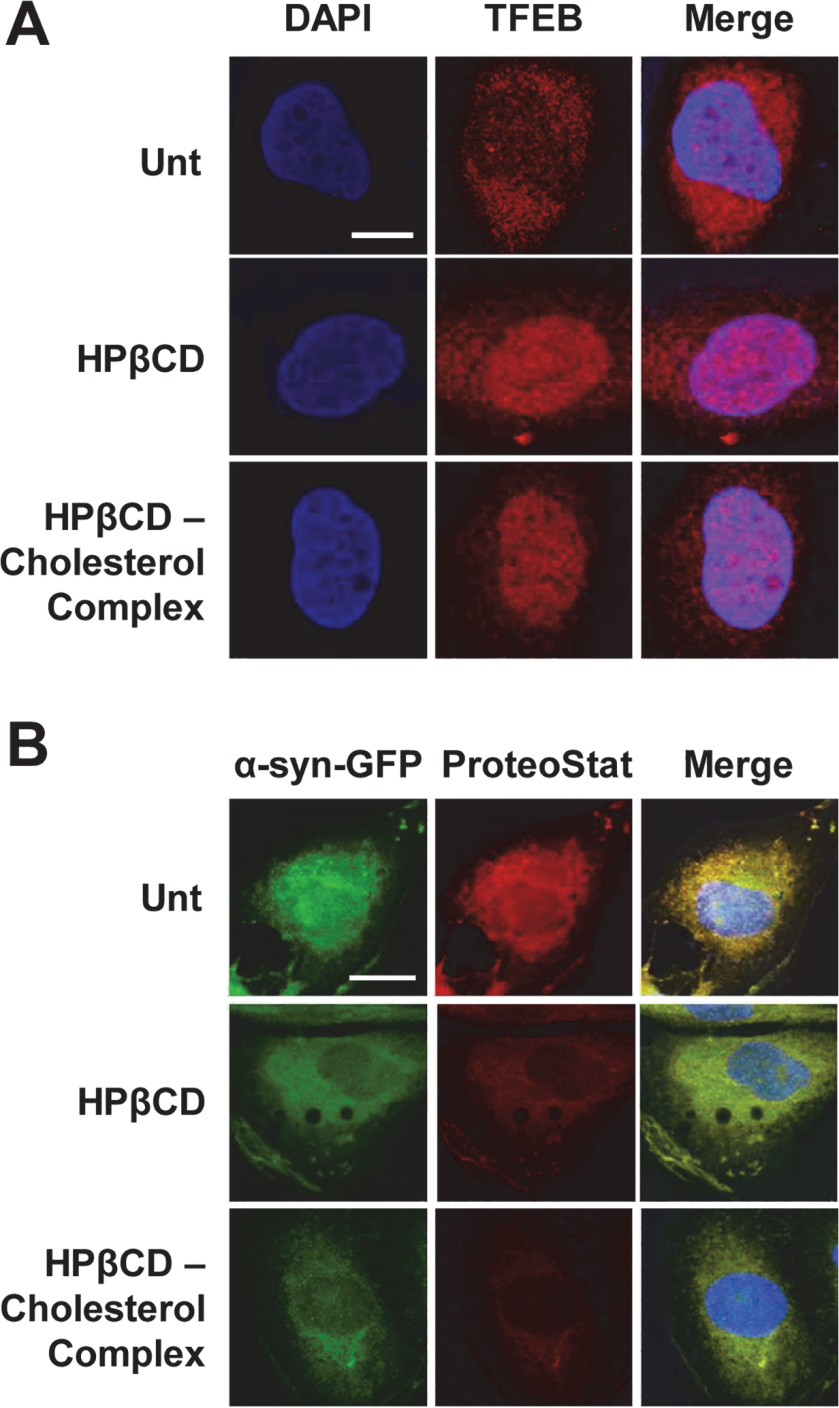
HPβCD-mediated clearance of α-syn aggregates does not depend on the ability of HPβCD to alter cholesterol levels. **a)** Immunofluorescence microscopy analyses of TFEB subcellular localization in H4/α-syn-GFP cells untreated or treated with HPβCD (1 mM) or HPβCD–cholesterol complexes (1 mM) for 24 h. TFEB nuclear localization was monitored using a FLAG-specific antibody and DAPI nuclear stain. Colocalization of DAPI (blue, column 1) and TFEB (red, column 2) is shown in purple (column 3). Representative images are reported. Scale bar represents 10 μm. **b)** Fluorescence microscopy analyses of H4/α-syn-GFP cells untreated or treated with HPβCD (1 mM) or HPβCD–cholesterol complex (1 mM) for 24 h. Images of α-syn-GFP fluorescence (green, column 1) and aggregates, detected using the ProteoStat dye (red, column 2), were merged (column 3) and analyzed using NIH ImageJ software. Representative images are reported. Scale bar represents 20 μm.

A number of studies demonstrate that cyclodextrins can alter the cellular concentration of cholesterol by extracting cholesterol from the plasma membranes [45,46] or by reducing lysosomal cholesterol content [47]. Cholesterol depletion from plasma membranes has been demonstrated to affect many cellular processes [48], particularly autophagy [49]. Therefore, we asked whether the reduction in α-syn aggregates observed in H4/α-syn-GFP cells treated with HPβCD is due to the ability of HPβCD to alter cellular levels of cholesterol. To address this question, we tested TFEB activation and the accumulation of α-syn aggregates in cells treated with HPβCD-cholesterol inclusion complexes. HPβCD-cholesterol complexes were prepared by saturating HPβCD with cholesterol as previously described [45]. H4/α-syn-GFP cells were treated with HPβCD (1 mM) or HPβCD-cholesterol complexes (1 mM) and TFEB subcellular localization was examined by confocal microscopy (Fig. 6A). Microscopy analyses revealed that HPβCD-cholesterol complexes induce nuclear translocation of TFEB and that the extent of nuclear translocation is comparable to that observed in cells treated with HPβCD that is not saturated with cholesterol. These results suggest that HPβCD-induced activation of TFEB in H4/α-syn-GFP cells is independent of HPβCD ability to deplete the intracellular levels of cholesterol.

To investigate whether the ability of HPβCD to deplete the intracellular levels of cholesterol affects clearance of α-syn, we evaluated the accumulation of α-syn aggregates in H4/α-syn-GFP cells treated with HPβCD or HPβCD-cholesterol complexes as described above (Fig. 6B). Cell treatment with HPβCD-cholesterol complexes resulted in reduction of α-syn aggregates as indicated by reduced binding of ProteoStat dye and lack of colocalization between GFP and ProteoStat dye signals. Moreover, cell treatment with HPβCD-cholesterol complexes resulted in reduction in α-syn aggregates to an extent comparable to that observed upon treatment with HPβCD. These results suggest that HPβCD-induced activation of TFEB and HPβCD-mediated clearance of α-syn aggregates in H4/α-syn-GFP cells does not depend on the ability of HPβCD to alter the intracellular concentration of cholesterol.

## Conclusions

The recent discovery of TFEB has provided a novel target for regulating clearance of autophagic cargo by the lysosome [27,50]. Indeed, TFEB was found to provide neuroprotection *in vivo* by restoring lysosomal function and enhancing autophagy [31,32]. We report here that genetic and chemical activation of TFEB promotes autophagic clearance of aggregated α-syn. While the studies reported herein focus primarily on clearance through macroautophagy, we do not exclude the possibility that TFEB-mediated degradation of misfolded α-syn directly by the lysosome could also contribute to the observed reduction in α -syn aggregates [10]. Nevertheless, results from this study identify TFEB as a therapeutic target to reduce the accumulation of α-syn aggregates and motivate the discovery of chemical activators of TFEB for therapeutic intervention.

The autophagy-activating properties of β-cyclodextrins (βCDs), a family of cyclic oligosaccharides known to deplete cholesterol from biological membranes have been previously reported [46,49] and shown to mediate clearance of aberrant storage material in lysosomal storage disorders [43,51]. In this study, HPβCD treatment was used to induce chemical activation of TFEB. The finding that HPβCD treatment mediates reduction in α-syn aggregates through activation of TFEB and upregulation of autophagy resonates with previously reported evidence demonstrating that MβCD treatment results in reduction of α-syn in cell and mouse model systems [44,52] and suggest that TFEB-mediated clearance of α-syn aggregates may play a key role in the mechanisms of βCD-induced neuroprotection observed in α-syn transgenic rats.

The notion that cell exposure to HPβCD results in enhanced autophagic clearance independent of HPβCD ability to alter the cellular pool of cholesterol suggests that induction of autophagy is likely linked to the adaptive cellular response that is activated upon internalization of HPβCD. βCDs enter the cell through endocytosis [47] and endocytic delivery of HPβCD to the lysosome was shown to alter lysosomal proteostasis [53]. These observations suggest a model in which TFEB is activated upon internalization of HPβCD to restore lysosomal proteostasis [29] and that this process likely to proceed via mTOR, a key regulator in the autophagy pathway that monitors the status of the lysosome and controls TFEB activation [54]. Activation of TFEB and upregulation of the autophagy-lysosomal system in response to HPβCD internalization, in turn, mediates clearance of α-syn aggregates. The activation of autophagic clearance mediated by HPβCD and consequent reduction in α-syn aggregates is likely to compensate the inefficient or impaired function of the UPS which is typically associated with the accumulation of misfolded and aggregated α-syn [6] and with the development of familial forms of PD [7].

In summary, we present evidence that genetic and chemical activation of TFEB reduces the accumulation of aggregated α-syn and promotes α-syn clearance by enhancing the autophagy pathway. This study also provides proof-of-principle evidence that chemical activation of TFEB is a viable therapeutic strategy to enhance the degradation of α-syn aggregates and motivates the discovery of alternative compounds that can effectively cross the blood-brain barrier [55] for the treatment of PD and potentially other neurodegenerative diseases characterized by protein deposition. Finally, results from this study describing the impact of HPβCD on the lysosome-autophagy system are likely to inform a variety of drug delivery applications in which βCD is routinely used as excipient to increase the solubility and bioavailability of drugs [56].

## Methods

### Reagents and Cell Cultures

2-hydroxypropyl-β-cyclodextrin (HPβCD) and cholesterol were purchased from Sigma-Aldrich, bafilomycin was from Cayman Chemical, and DAPI nuclear stain was from Enzo Life Sciences. TFEB siRNA (Cat. No. SI00094969) and control siRNA (Cat. No. 1027280) were purchased from Qiagen. pMSCV-PIG, gag-pol, and VSVG plasmids were from Addgene and TFEB-3XFLAG plasmid was a generous gift from Dr. Marco Sardiello (Baylor College of Medicine, Houston, TX)

H4 cells stably transfected for the expression of α-syn-EmGFP (H4/α-syn-GFP) were generated as previously described [34,35]. Human H4 neuroglioma (HTB-148, ATCC), H4/α-syn-GFP, and HEK-293T cells (CRL-11268, ATCC) were cultured in high glucose DMEM (Fisher Scientific) supplemented with 10% fetal bovine serum and 1% PSQ, and maintained at 37°C and 5% CO_2_.

### TFEB Retrovirus Plasmid Construction and Transduction

The plasmid, pMSCV-PI650/TFEB, was constructed as follows: first, the GFP cassette in the pMSCV-PIG plasmid was replaced with eqFP650 using NcoI and SalI restriction enzyme sites creating pMSCV-PI650. TFEB-3XFLAG was inserted into the MCS of pMSCV-PI650 using BglII and XhoI generating pMSCV-PI650/TFEB. pMSCV-PI650/TFEBS142A was obtained by site directed mutagenesis of pMSCV-PI650/TFEB using a reverse primer containing the S142A point mutation, 5’–TGGCCATGGGAGCATTGGGAGCAC—3’.

Retrovirus particles were generated as follows: HEK-293T cells were cultured in 10 cm dishes and transfected with 10 μg of pMSCV-PI650/TFEB or pMSCV-PI650/TFEB-S142A and 5 μg each of plasmids expressing the helper genes, gag-pol and VSVG, using Lipofectamine 2000 according to manufacturer’s instructions (Invitrogen). After 8 h, the transfection medium was replaced with fresh medium and incubated for 48 h. Retrovirus particles were collected by removing the medium using a sterilized syringe and filtered with 0.45 um syringe filter. Polybrene (8 μg/ml) was added to the retrovirus before transducing cells.

Retroviral gene transduction experiments were conducted as follows: H4/α-syn-GFP cells were plated in 6-well plates at a concentration of 5 x 10^4^ cells/ml and cultured overnight. The medium was removed and replaced with medium containing retrovirus particles and the plates were centrifuged at 2500 rpm for 90 min at 30°C. Cells were incubated at 37°C for 24–48 h before analysis.

### Aggregation Studies

α-syn-GFP aggregation was measured using the ProteoStat Aggregation detection kit (Enzo Life Sciences) according to manufacturer’s protocol. Fluorescence microscopy analyses were conducted as previously described [35] using a FluoView FV1000 (Olympus, 60X). Colocalization of α-syn-GFP and the ProteoStat dye in H4/α-syn-GFP cells was evaluated using the Colocalization Colormap script, an ImageJ plugin that calculates the correlation of intensity between complementary fluorescent signals [35].

Flow-cytometry analyses to quantify total cellular protein aggregation were conducted as previously described [35]. The aggregation propensity factor (APF) using the following formula: APF = 100 x (MFI_treated_—MFI_control_)/MFI_treated_ where MFI is the mean fluorescence intensity of the ProteoStat dye and a sample of untreated cells was used as control. Fluorescence intensity was measured by flow cytometry (FACSCanto II, BD Biosciences) using a 488-nm argon laser. APF was corrected for TFEB expression level (evaluated by qRT-PCR) to eliminate differences in ProteoStat binding due to the variability of transduction efficiency.

### Immunofluorescence Studies

Immunofluorescence studies were conducted as previously described [43]. Briefly, cells were cultured on acid-washed glass coverslips. After cell transduction (conducted as described above) or cell treatment with HPβCD (1 mM), cells were fixed using 4% paraformaldehyde, permeabilized with 0.1% Triton X-100, and incubated with 8% bovine serum albumin. To detect TFEB nuclear localization, cells were incubated with rabbit anti-TFEB antibody (Genetex) overnight at 4°C, washed with 0.1% Tween/PBS, and incubated for 1 h with DyLight 549 goat anti-rabbit antibody (Rockland Immunochemical). To detect LC3-LAMP2 colocalization, cells were incubated with rabbit anti-LC3 antibody (MBL International) and mouse anti-LAMP2 antibody (BioLegend) for 1 h, washed, and incubated with DyLight 549 goat anti-rabbit antibody and DyLight 649 goat anti-mouse antibody (Rockland Immunochemical) for 1 h. Images were collected at 60X using a confocal microscope (FluoView FV1000, Olympus) and analyzed using NIH ImageJ software.

Colocalization of LC3 and LAMP2 was quantified using Matlab. The background signal was subtracted from LC3 and LAMP2 images by removing red (LC3) and blue (LAMP2) pixels that displayed a brightness signal below a predefined threshold. To ensure that both fluorescent signals are within the same order of magnitude, LC3 and LAMP2 pixels that present brightness signal within a predefined range (0.5 and 2) were designated as positive correlation and selected to calculate the percentage of colocalziation. The percentage of colocalization was calculated by normalizing the number of pixels presenting LC3 and LAMP2 positive correlation by the total number of pixels in each cell over the entire image. Average values were calculated over multiple images and replicate samples.

### siRNA Transfection

siRNA transfection was performed using HiPerFect transfection reagent (Qiagen) as previously described [29]. Each well of a 6-well plate was seeded with 150 ng of siRNA in 25 μl of RNase-free water. 12 μl of HiPerFect transfection reagent were diluted with 63 μl of serum-free culture medium, added to each well and incubated for 10 min at room temperature. A 2 ml-solution of medium containing 8 x 10^4^ cells was added to each well and the plates were incubated at 37°C for 48 h. The medium was replaced with fresh medium or fresh medium containing 1 mM HPβCD and microscopy analyses were preformed after 24 h of treatment.

### Quantitative RT-PCR

RT-PCR analyses was conducted as previously described [35] using the primers reported in the S1 Table. Total RNA was extracted using RNAGEM Tissue reagent (ZyGEM). cDNA was synthesized from total RNA using qScript cDNA SuperMix (Quanta Biosciences) and quantified using a NanoDrop (Thermo Scientific). Quantitative PCR reactions were performed using PerfeCTa SYBR Green FastMix (Quanta Biosciences) in a CFX96 Real-Time PCR Detection System (Bio-Rad) with corresponding primers in S1 Table online. Samples were heated for 2 min at 95°C and amplified using 45 cycles of 1 s at 95°C, 30 s at 60°C, and 30 s at 72°C. Analyses were conducted using CFX Manager software (Bio-Rad) and the threshold cycle (C_T_) was extracted from the PCR amplification plot. The ΔC_T_ value was used to describe the difference between the C_T_ of a target gene and the C_T_ of the housekeeping genes, *GAPDH* and *ACTB*. The relative mRNA expression level of treated cells was normalized to that of untreated cells: relative mRNA expression level = 2 exp [−(ΔC_T_ (treated cells) – ΔC_T_ (untreated cells))]. Each data point was evaluated in triplicate and measured three times.

### Cell Fractionation

Cell fractionation experiments were conducted as previously described [34,35]. Briefly, H4/α-syn-GFP cells were plated in 10-cm culture dishes at a concentration of 1.0 x 10^5^ cells/mL and treated with HPβCD (1 mM, 3mM and 5mM) for 24 h. The soluble protein fraction was extracted by resuspending the cells in Complete Lysis-M Buffer (Roche) supplemented with 1% Triton X-100 and incubating on ice with gentle agitation for 30 min followed by centrifugation at 15,000 x g for 60 min at 4°C. The pellet was resuspended in Complete Lysis-M Buffer supplemented with 2% SDS and 8M urea and sonicated to collect the insoluble protein fraction. Protein concentrations were determined by Bradford assay, and samples were diluted to the same concentration and separated by 12% SDS-PAGE. Western blot analyses were performed using mouse anti-α-syn (Sigma), and rabbit anti-GAPDH (Santa Cruz) antibodies and appropriate secondary antibodies (HRP conjugated anti-mouse (Stressgen), and anti-rabbit (Santa Cruz)). Blots were visualized using Luminata Forte Western HRP Substrate (Millipore) and bands were quantified with NIH ImageJ software.

### Western blot analyses

H4/α-syn-GFP cells were plated in 10-cm culture dishes at a concentration of 1.0 x 10^5^ cells/ml and treated with HPβCD (1 mM) for 48 h. The total protein content was extracted by incubating the cells in Complete Lysis-M buffer (Roche) according to manufacturer’s protocol. Protein concentrations were determined by Bradford assay; samples were diluted to the same concentration and separated by gel filtration using a 15% SDS-PAGE gel. Western blot analyses were performed using rabbit anti-LC3 (Sigma) and rabbit anti-GAPDH (Santa Cruz) antibodies and appropriate secondary antibodies (HRP conjugated anti-rabbit (Santa Cruz)). Blots were visualized using Luminata Forte Western HRP Substrate (Millipore) and bands were quantified with NIH ImageJ software.

### Preparation of HPβCD–Cholesterol Complexes

HPβCD–cholesterol complexes were prepared as previously described [57]. 10 μl of cholesterol from a stock solution of 50 μg/mL prepared in chloroform:methanol 1:1 (v:v), was added to a glass tube and the solvent was evaporated under a gentle stream of nitrogen. 10 ml of 10 mM HPβCD dissolved in DMEM medium without serum was added to the dried cholesterol and the solution was sonicated in a bath sonicator for 3 min followed by overnight incubation in a rotating water bath at 37°C. The solution was diluted to 1mM in DMEM and filtered through a 0.45 μm syringe filter to remove excess cholesterol crystals immediately prior to adding the HPβCD–cholesterol complexes to the cells.

### Toxicity Assay

Induction of apoptosis was measured as previously described [35]. H4/α-syn-GFP cells were treated with taxol (50 nM) and HPβCD (1 mM) for 16 h at 37°C. Cells were collected and resuspended in 100 μL of 1X binding buffer (BD Biosciences). Samples were incubated with 5 μL of Annexin V-Cy5 (BD Biosciences) and 5 μl of PI (BioLegend) for 20 min in the dark at room temperature. Samples were diluted with 400 μL 1X binding buffer and analyzed by flow cytometry (FACSCanto II, BD Biosciences) with a 533-nm Helium Neon laser for Cy5 fluorescence and 488-nm Argon laser for PI fluorescence.

### Statistical Analysis

All data are presented as the mean ± SD, and statistical significance was calculated using a two-tailed t-test.

## Supporting Information

S1 Fig Overexpression of TFEB upregulates the CLEAR network in H4/α-syn-GFP cells. Relative mRNA expression levels of representative CLEAR network genes in H4/α-syn-GFP cells transduced to express TFEB-3xFLAG or S142A TFEB-3xFLAG. *GBA*, *HEXA*, and *LAMP1* mRNA expression levels were obtained by qRT-PCR, corrected for the expression of the housekeeping genes, *GAPDH* and *ACTB*, and normalized to those of untreated cells (dashed line). Data are reported as the mean ± SD (n≥3; p < 0.05).
(TIF)

S2 Fig HPβCD does not induce cytotoxicity in H4/α-syn-GFP cells. Relative Annexin V-binding affinity **(A)** and PI population **(B)** in H4/α-syn-GFP cells treated with taxol (25 nM, used here as control) and HPβCD (1 mM) for 16 h. Data are reported as the mean ± SD (n≥3; p < 0.05).
(TIF)

S3 Fig Treatment with control siRNA does not alter HPβCD-induced reduction in α-syn aggregates. Fluorescence microscopy analyses of H4/α-syn-GFP cells treated with control siRNA and HPβCD (1 mM) for 24 h. Images of α-syn-GFP fluorescence (green, column 1) and aggregates, detected using the ProteoStat dye (red, column 2), were merged (column 3) and analyzed using NIH ImageJ software. Scale bar represents 20 μm.
(TIF)

S4 Fig Western blot analyses of α-syn in Triton X-100 insoluble fractions of H4/α-syn-GFP cells treated with HPβCD (1mM, 3mM and 5mM) for 24 h. Western blot images were analyzed using NIH ImageJ software, and the amount of insoluble α-syn was normalized to that of untreated cells. Data are reported as the mean ±SE (*p < 0.05)
(TIF)

S1 Table Primer Sequences Used in Quantitative RT-PCR. (DOCX)
