# Supplementary figures and images for: Genetic and Chemical Activation of TFEB Mediates Clearance of Aggregated α-Synuclein
Source: PLoS One. 2015 Mar 19;10(3):e0120819. doi: 10.1371/journal.pone.0120819 (PMC4366176; doi:10.1371/journal.pone.0120819)

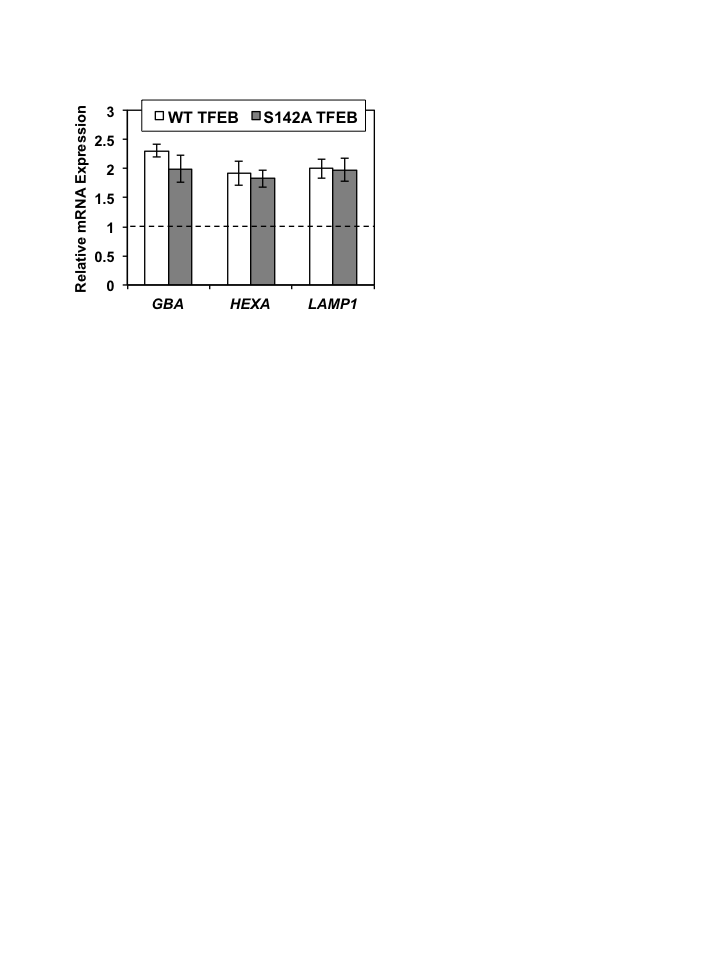

Supplement: S1 Fig — Relative mRNA expression levels of representative CLEAR network genes in H4/α-syn-GFP cells transduced to express TFEB-3xFLAG or S142A TFEB-3xFLAG. GBA, HEXA, and LAMP1 mRNA expression levels were obtained by qRT-PCR, corrected for the expression of the housekeeping genes, GAPDH and ACTB, and normalized to those of untreated cells (dashed line). Data are reported as the mean ± SD (n≥3; p < 0.05). (TIF) [file pone.0120819.s001.tif]

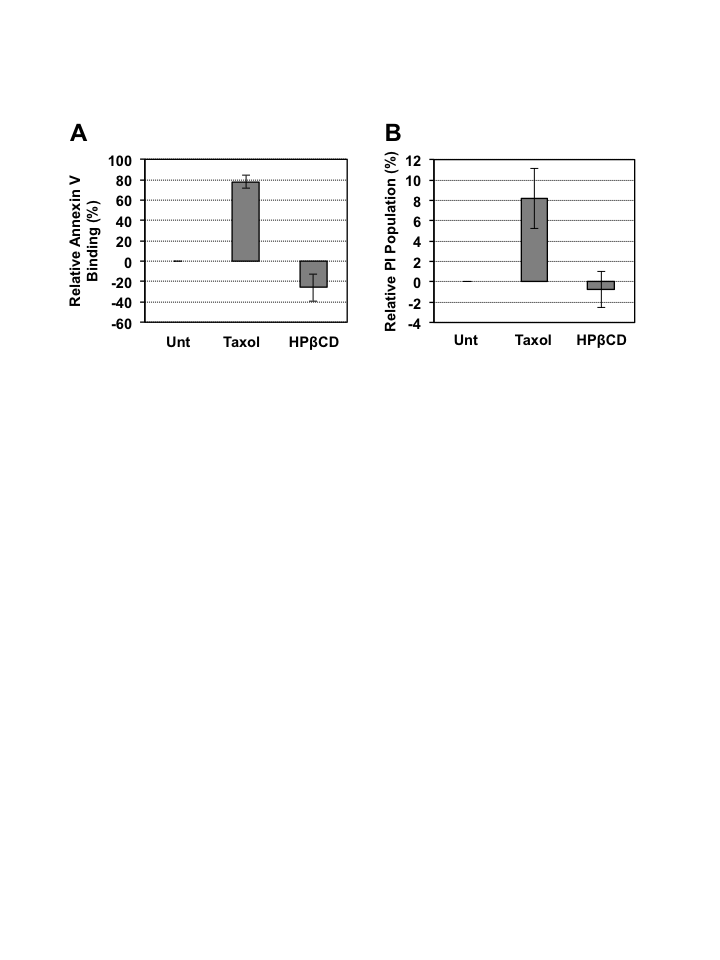

Supplement: S2 Fig — Relative Annexin V-binding affinity (A) and PI population (B) in H4/α-syn-GFP cells treated with taxol (25 nM, used here as control) and HPβCD (1 mM) for 16 h. Data are reported as the mean ± SD (n≥3; p < 0.05). (TIF) [file pone.0120819.s002.tif]

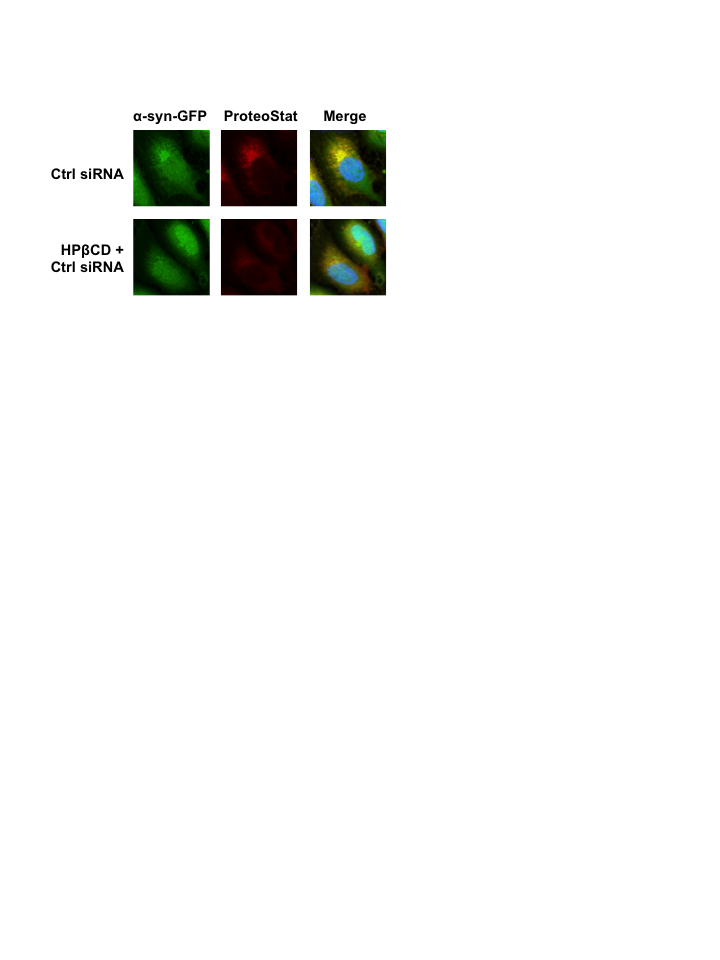

Supplement: S3 Fig — Fluorescence microscopy analyses of H4/α-syn-GFP cells treated with control siRNA and HPβCD (1 mM) for 24 h. Images of α-syn-GFP fluorescence (green, column 1) and aggregates, detected using the ProteoStat dye (red, column 2), were merged (column 3) and analyzed using NIH ImageJ software. Scale bar represents 20 μm. (TIF) [file pone.0120819.s003.tif]

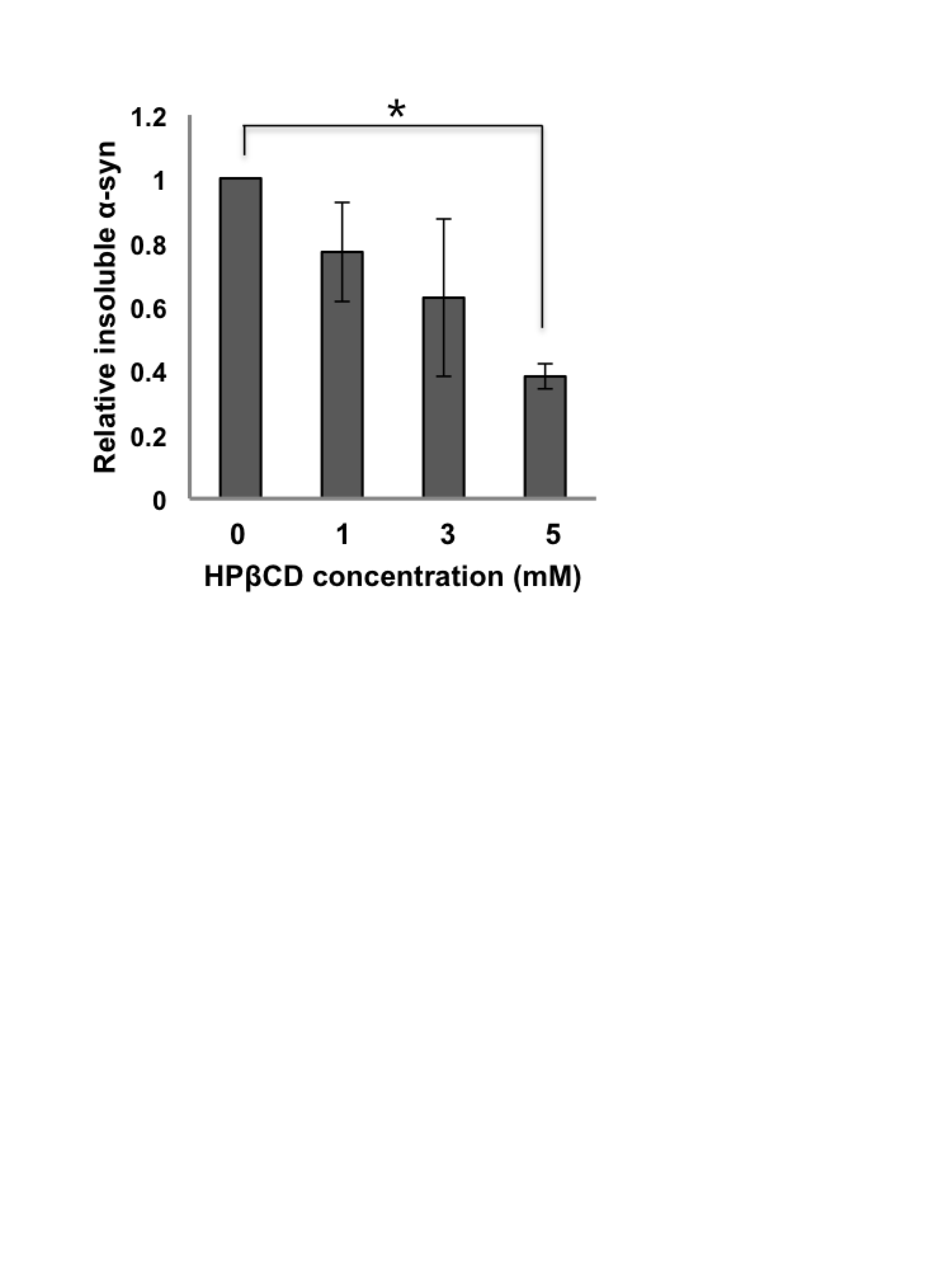

Supplement: S4 Fig — Western blot images were analyzed using NIH ImageJ software, and the amount of insoluble α-syn was normalized to that of untreated cells. Data are reported as the mean ±SE (*p < 0.05) (TIF) [file pone.0120819.s004.tif]
