# Supplementary material for: Genetic and Chemical Activation of TFEB Mediates Clearance of Aggregated α-Synuclein
Source: PLoS One. 2015 Mar 19;10(3):e0120819. doi: 10.1371/journal.pone.0120819 (PMC4366176; doi:10.1371/journal.pone.0120819)
Supplement: S1 Table — (DOCX) [file pone.0120819.s005.docx]

# Genetic and chemical activation of TFEB mediates clearance of aggregated α-synuclein

Kiri Kilpatrick^1^, Yimeng Zeng^1^, Tommy Hancock^1^, and Laura Segatori^1,2,3*^

Departments of ^1^Chemical and Biomolecular Engineering, ^2^Bioengineering, and ^3^Biochemistry and Cell Biology. Rice University, Houston, Texas, United States

*Correspondence: [segatori@rice.edu](mailto:segatori@rice.edu), 713-348-3536

## Supplementary Figure Legends

S1 Figure. Overexpression of TFEB upregulates the CLEAR network in H4/α-syn-GFP cells. Relative mRNA expression levels of representative CLEAR network genes in H4**/**α-syn-GFP cells transduced to express TFEB-3xFLAG or S142A TFEB-3xFLAG. *GBA,* *HEXA,* and *LAMP1* mRNA expression levels were obtained by qRT-PCR, corrected for the expression of the housekeeping genes, *GAPDH* and *ACTB*, and normalized to those of untreated cells (dashed line). Data are reported as the mean ± SD (n≥3; p < 0.05).

S2 Figure. **HPβCD** does not induce cytotoxicity in H4/α-syn-GFP cells. Relative Annexin V-binding affinity **(A)** and PI population **(B)** in H4/α-syn-GFP cells treated with taxol (25 nM, used here as control) and HPβCD (1 mM) for 16 h. Data are reported as the mean ± SD (n≥3; p < 0.05).

S3 Figure. Treatment with control siRNA does not alter **HPβCD**-induced reduction in α-syn aggregates. Fluorescence microscopy analyses of H4/α-syn-GFP cells treated with control siRNA and HPβCD (1 mM) for 24 h. Images of α-syn-GFP fluorescence (green, column 1) and aggregates, detected using the ProteoStat^®^ dye (red, column 2), were merged (column 3) and analyzed using NIH ImageJ software. Scale bar represents 20 µm.

S4 Figure. Western blot analyses of α-syn in Triton X-100 insoluble fractions of H4/α-syn-GFP cells treated with HPβCD (1mM, 3mM and 5mM) for 24 h. Western blot images were analyzed using NIH ImageJ software, and the amount of insoluble α-syn was normalized to that of untreated cells. Data are reported as the mean ±SE (*****p < 0.05)

## Supplementary Tables

### S1 Table. Primer Sequences Used in Quantitative RT-PCR.

| Gene | GenBank Accession Code | Forward Primer (5’ → 3’) | Reverse Primer (5’ → 3’) | Reference |
| --- | --- | --- | --- | --- |
| *ACTB* | NM_001101 | gatcattgctcctcctgagc | ACTCCTGCTTGCTGATCCAC | [1](#_ENREF_1) |
| *GAPDH* | NM_002046 | GTCGGAGTCAACGGATT | AAGCTTCCCGTTCTCAG | [2](#_ENREF_2) |
| *TFEB* | NM_007162 | CCAGAAGCGAGAGCTCACAGAT | TGTGATTGTCTTTCTTCTGCCG | [3](#_ENREF_3) |
| *GBA* | NM_000157 | CCAAGCCTTTGAGTAGGGTAAG | CCCGTGTGATTAGCCTGGAT | [3](#_ENREF_3) |
| *HEXA* | NM_000520 | CAACCAACACATTCTTCTCCA | CGCTATCGTGACCTGCTTTT | [3](#_ENREF_3) |
| *LAMP1* | NM_005561 | ACGTTACAGCGTCCAGCTCAT | TCTTTGGAGCTCGCATTGG | [3](#_ENREF_3) |
| *BECN1* | NM_003766 | GGCTGAGAGACTGGATCAGG | CTGCGTCTGGGCATAACG | [4](#_ENREF_4) |
| *MAPLC3* | NM_181509 | GAGAAGCAGCTTCCTGTTCTGG | GTGTCCGTTCACCAACAGGAAG | [4](#_ENREF_4) |
| *SQSTM1* | NM_003900 | GCACCCCAATGTGATCTGC | CGCTACACAAGTCGTAGTCTGG | [4](#_ENREF_4) |
| *UVRAG* | NM_003369 | ATGCCAGACCGTCTTGATACA | TGACCCAAGTATTTCAGCCCA | [4](#_ENREF_4) |
